# Vaccine Strain-Specificity of Protective HLA-Restricted Class 1 *P*. *falciparum* Epitopes

**DOI:** 10.1371/journal.pone.0163026

**Published:** 2016-10-03

**Authors:** Martha Sedegah, Bjoern Peters, Michael R. Hollingdale, Harini D. Ganeshan, Jun Huang, Fouzia Farooq, Maria N. Belmonte, Arnel D. Belmonte, Keith J. Limbach, Carter Diggs, Lorraine Soisson, Ilin Chuang, Eileen D. Villasante

**Affiliations:** 1 Malaria Department, Naval Medical Research Center, Silver Spring, MD, 20910, United States of America; 2 La Jolla Institute for Allergy and Immunology, La Jolla, CA, 92037, United States of America; 3 Henry M. Jackson Foundation for the Advancement of Military Medicine, Rockville, MD, 20817, United States of America; 4 USAID, Washington, DC, 20523, United States of America; Institut de recherche pour le developpement, FRANCE

## Abstract

A DNA prime/adenovirus boost malaria vaccine encoding *Plasmodium falciparum* strain 3D7 CSP and AMA1 elicited sterile clinical protection associated with CD8+ T cell interferon-gamma (IFN-γ) cells responses directed to HLA class 1-restricted AMA1 epitopes of the vaccine strain 3D7. Since a highly effective malaria vaccine must be broadly protective against multiple *P*. *falciparum* strains, we compared these AMA1 epitopes of two P. *falciparum* strains (7G8 and 3D7), which differ by single amino acid substitutions, in their ability to recall CD8+ T cell activities using ELISpot and flow cytometry/intracellular staining assays. The 7G8 variant peptides did not recall 3D7 vaccine-induced CD8+ T IFN-γ cell responses in these assays, suggesting that protection may be limited to the vaccine strain. The predicted MHC binding affinities of the 7G8 variant epitopes were similar to the 3D7 epitopes, suggesting that the amino acid substitutions of the 7G8 variants may have interfered with TCR recognition of the MHC:peptide complex or that the 7G8 variant may have acted as an altered peptide ligand. These results stress the importance of functional assays in defining protective epitopes.

***Clinical Trials Registrations*:** NCT00870987, NCT00392015

## Introduction

Recently we demonstrated that a heterologous DNA-prime/human adenovirus 5 (HuAd5) boost vaccine encoding two *Plasmodium falciparum* 3D7 strain antigens, circumsporozoite protein (CSP) and apical membrane antigen-1 (AMA1), induced sterile protection against controlled human malaria infection (CHMI) in four of 15 immunized subjects [1]. Protection was associated with ELISpot and CD8+ T cell interferon-gamma (IFN-γ) responses to AMA1 using peripheral blood mononuclear cells (PBMC) taken just prior to CHMI [1]. Without DNA-priming the HuAd5 vaccine alone did not elicit sterile protection, but often elicited similar or higher ELISpot and CD8+ T cell IFN-γ responses to CSP and AMA1 than the protected subjects [2].

We sought to further investigate these differences and found that quality rather than quantity of CD8+ T cell responses were crucial. We used 12 peptide pools containing 15mer peptides spanning the entire sequence of AMA1, and found that responses of three of the four protected subjects were narrowly focused on discrete regions of AMA1 represented by single peptide pools, designated Ap8 and Ap10, whereas responses of non-protected subjects from the DNA/HuAd5 and HuAd5 alone trials were more broadly reactive to multiple regions of AMA1 [3]. Activities of protected subjects to Ap8 or Ap10 represented a higher percent of the total response to all peptide pools than non-protected subjects to Ap8 or Ap10 [3]. We suggested that these focused responses were genetically-restricted as the protected subjects recognized single 15mer peptides within Ap8 and Ap10, and these 15mers also recalled ELISpot and CD8+ T cell IFN-γ responses from these subjects.

The NetMHC [4] algorithm which predicts peptide binding to MHC class I molecules in terms of 50% inhibitory concentration (IC_50_) values was used to predict putative 3D7 AMA1 class I epitopes within these 15mers. Experimental testing of peptides representing these predicted epitopes indeed recalled T cell responses from protected subjects. Ap10 contained the predicted HLA A*11:01-restricted epitope NSTCRFFVCK that recalled responses from one DNA/Ad-protected subject (v11) expressing HLA A*11:01 (HLA supertype A*03), and Ap8 contained the predicted HLA B*57:01/B*58:01 epitope KSHGKGYNW that recalled responses from the two DNA/Ad-protected subjects (v10 and v18) expressing HLA B*57:01 or B*58:01 (HLA B*58 supertype) [3]. Among the non-protected subjects in the HuAd5 trial, one subject (v194) who expressed HLA B*58:01 and recognized the B*58 epitope showed a significant delay to patency (suggesting reduction in numbers of liver stage parasites), suggesting partial protection [3]. The fine specificity of the HLA expressed by protected and non-protected subjects was crucial as two non-protected subjects (v135, and v179) from the HuAd5 alone trial also strongly recognized the same protection associated epitope within Ap8 but expressed A*32:01 (HLA supertype A*01), and two non-protected subjects (v126, and v172) from the HuAd5 trial recognized the same protection associated epitope within Ap10 but expressed HLA A*30:01 or A*03:01 (HLA A*01A03 and A*03 supertypes). We suggested a hypothesis that vaccine-induced effector memory CD8+ T cells recognizing a single class I HLA A*03 or HLA B*58 epitope can confer protection to *P*. *falciparum* in humans [3].

The 3D7 strain of *P*. *falciparum* was used for both immunization and CHMI in the DNA/HuAd5 and HuAd5 trials [1, 2]. Since *P*. *falciparum* is genetically diverse [5], and AMA1 is polymorphic due entirely to single amino acid substitutions [6], a major question is whether a *P*. *falciparum* 3D7 DNA/HuAd5 vaccine would elicit protective epitope-specific responses to genetically-diverse *P*. *falciparum* strains in areas of natural transmission. The protective 3D7 A*03 (NSTC**R**FFVCK) and B*58 (KS**H**GKGYNW) epitopes each contain a dimorphic residue that occur in the 7G8 strain A*03 epitope (NSTC**N**FFVCK) and 7G8 B*58 epitope (KS**R**GKGYNW) [7]. The objective of this study was to determine whether subjects immunized with 3D7 AMA1 using the DNA/HuAd5 [1] and HuAd5 alone [2] regimens recognized the 7G8 dimorphic variant epitopes.

One potential constraint to these studies was the small numbers of vaccine immunized subjects and availability of PBMC from these subjects, particularly prior to CHMI. We have previously used post-CHMI PBMCs in ELISpot IFN-γ assays as the AMA1 peptide pool specificities were maintained even though the magnitudes of responses were reduced [3]. However, we were able to use PBMCs from pre-CHMI of protected subjects in ICS assays to confirm the ELISpot outcomes. As before [3], these results are reported with the proviso that post-CHMI cells may not fully reflect pre-CHMI activities.

## Materials and Methods

### Ethics

The study protocol was reviewed and approved by the NMRC Institutional Review Board in compliance with all federal regulations governing the protection of human subjects. WRAIR holds a Federalwide Assurance from the Office of Human Research Protections (OHRP) under the Department of Health and Human Services as does NMRC. All key personnel were certified as having completed mandatory human research ethics education curricula and training under the direction of the WRAIR IRB or the NMRC Office of Research Administration (ORA) and Human Subjects Protections Program (HSPP). All potential study subjects provided written, informed consent before screening and enrollment and had to pass an assessment of understanding.

### Human subjects used in this study

Informed consent was obtained from all subjects in these trials. Protected and non-protected subjects in the 3D7 CSP and AMA1 DNA/HuAd5 [1] and non-protected subjects in the HuAd5 trial [2], who were high responders to Ap8 and Ap10 [3], are shown in Table 1. Peripheral blood mononuclear cells (PBMCs) from the DNA/HuAd5 vaccine were collected 22/23 days post immunization one week before CHMI, or 12 weeks post CHMI when activities were lower but represented pre-CHMI activities (Table 1) [3]; PBMCs from HuAd5 immunized subjects were taken four or 12 weeks post-CHMI when activities were lower but also represented pre-CHMI activities, as shown in Table 1 [2].

**Table 1:** Immunization, protection and previously reported immunogenicity of human subjects used in this study.

Vaccine	Subject	Status	HLA	HLA	ELISpot (sfc/m)							
			alleles	supertype	Ap8				Ap10			
					sfc/m	#+	sfc/m	#+	sfc/m	#+	sfc/m	#+
**DNA/HuAd5**	v10	Protected	A*01:01; A*26:01	B*58	**567**^1^	2	**196**^2^	1	3^1^	2	1^2^	2
			B*44:02; B*57:01									
	v11	Protected	A*11:01; A*68:02	A*03	13^1^	4	11^2^	2	**690**^1^	4	**360**^2^	2
			B*50:01; B*55:01									
	v18	Protected	A*02:01; A*02:05	B*58	**1024**^1^	2	**324**^2^	3	0^1^	2	4^2^	3
			B*58:01; B*58:01									
	v15	Non-Prot.	A*29:02; A*30:02	B*58	**143**^1^	1	35^3^	0	0^1^	1	0^3^	0
			B*15:02; B*57:01									
**HuAd5**	v125	Non-Prot.	A*02:01; A*11:01	A*03	**89**^1^	6	**108**^2^	6	**134**^1^	6	**104**^2^	6
			B*35:01; B*37:01									
	v126	Non-prot.	A*01:01; A*30:01	A*01A03	**163**^1^	7	**240**^2^	7	**278**^1^	7	**127**^2^	7
			B*13:02; B*37:01									
	v135	Non-prot.	A*24:01; A*32:01	A*01	**684**^1^	4	**73**^2^^,^^4^	2	4^1^	7	8^2^^,^^4^	2
			B*50:02; B*52:01									
	v149	Non-prot.	A*02:01; A*11:01	A*03	19^1^	4	30^2^	4	**423**^1^	4	**238**^2^	4
			B*44:02; B*55:01									
	v172	Non-prot.	A*01:01; A*03:01	A*03	**166**^1^	6	34^2^	5	**119**^1^	6	73^2^	5
			B*08:01; B*35:01									
	v179	Non-prot.	A*24:02; A*32:01	A*01	**774**^1^	7	**134**^3^	3	16^1^	7	26^3^	3
			B*15:01; B*39:06									
	v194	Non-prot.*	A*32:01; A*68:01	B*58	**839**^1^	4	**95**^3^	3	6^3^	4	1^4^	3
			B*13:02; B*58:01									

Non-prot. = non-protected; sfc/m = spot forming cells/million PBMCs

#+ number of the 12 tested AMA1 peptide pools that were positive. Positive ELISpot activities are shown in bold. Responses of protected subjects to Ap8 or Ap10 represented a higher percent of the total response to all peptide pools than non-protected subjects. Human subjects with the highest pre-CHMI activities to Ap8 or Ap10 were selected (ref. #3). Positive ELISpot activities are shown in bold. ELISpot IFN-γ activities of fresh PBMCs:

^1^22/23 days post HuAd5 boost/five or six days pre-CHMI

^2^four weeks after CHMI

^3^12 weeks after CHMI.

^4^This time point used cryopreserved PBMCs.

*Time to patency was significantly delayed but subject was not sterilely protected.

### Peptides

Full length 3D7 AMA1 was covered by 15mer peptides that were combined into 12 pools (Ap1-Ap12) each containing 10–13 peptides [8]. Ap8 contained thirteen 15mer peptides spanning AMA1 amino acids (aa) 365–427, and Ap10 contained thirteen 15mer peptides spanning aa 469–531 [8]. AMA1 15mer peptide pools Ap8 and Ap10 were used to recall responses from protected subjects as previously described [3]. AMA1 class 1 peptides were 3D7 A*03 (NSTC**R**FFVCK containing predicted epitope STC**R**FFVCK) or B*58 (KS**H**GKGYNW); 7G8 A*03 (NSTC**N**FFVCK containing predicted epitope STC**N**FFVCK) or B*58 (KS**R**GKGYNW). The NetMHC algorithm [9] was used to predict binding affinities (nM) of the 3D7 and 7G8 epitopes.

### *Ex vivo* Enzyme Linked Immunospot Interferon-gamma Assays (ELISpot IFN-γ)

Antigen-specific responses of previously cryopreserved PBMCs were evaluated in triplicate experiments as previously described [8, 10] Briefly, cryopreserved PBMC at 500K suspended in 100 μL complete medium were stimulated with AMA1 15mer peptide pools Ap8 or Ap10, or 3D7 or 7G8 AMA1 epitopes, at 10 μg/mL each peptide. The positive control was CEF-Class I Peptide Pool *Plus* (CTL, Ohio, USA). Results, expressed as spot forming cells/million PBMC (sfc/m), are shown as the magnitudes of triplicate responses of each tested subject to individual Ap8 or Ap10 peptide pools or 3D7 and 7G8 epitope peptides. A positive response (mean of triplicate experiments) was statistically defined (student t-test) as the significance of the difference with medium-only controls [3].

### ELISpot CD4+ and CD8+ depletion studies

Characterization of ELISpot IFN-γ-producing cells were performed with PBMC after depletion of T cell subsets using anti-human CD4+ and CD8+ coated Dynabeads (Dynal, Great Neck, NY) as previously described [3]. Assays were performed in duplicate or triplicate using 400K PBMC after depletion. The data is presented as the activities (sfc/m) of duplicate or triplicate wells after T cell subset depletion.

### Flow cytometry with intracellular staining (ICS)

Previously frozen PBMC were stimulated in duplicate experiments as previously described [8, 11]. PBMCs were suspended at 10x10^6^ cells/mL, in supplemented complete RPMI-1640 (BioWhittaker, Walkersville, MD) that contained 20% fetal calf serum (Hyclone, Logan, UT). AMA1 peptide pools Ap8 and Ap10 were tested at 10 μg/mL for each peptide, and 3D7 and 7G8 epitopes were tested at 10 μg/mL; control stimulants were medium alone and the CEF peptide pool (CTL, Ohio, USA). Cells were phenotyped as CD4+ and CD8+ T cells and were stained for IFN-γ. Stimulants were added to cells and incubated at 37°C with 5% CO_2_ for 2 hr. Brefeldin A (BD Bioscience) was added at a final concentration of 0.6 μL/mL and incubated at 37°C with 5% CO_2_ for approximately 12–15 hr. Cells were permeabilized with Cytofix/Cytoperm solution (BD Bioscience) and stained with anti-CD3 AlexaFluor700, anti-CD4+ PerCP, anti-CD8+ Pacific Blue, anti-IFN FITC, incubated and washed. Cells were resuspended and acquired on a BD LSRII using FACSDiVA (BD BioScience) software. 7-color flow cytometry was performed as previously described [8]. Data for peptide pools and individual peptides are corrected for medium only responses. A positive response was defined as the geometric mean of duplicate experiments that exceeded the geometric mean + 3 standard deviations of the medium-stimulated controls (>0.03%) [3]. The data of replicate experiments is presented as percent of total CD8+ T cells producing IFN-γ.

### Statistical analyses

Repeated measure analysis of variance was used to evaluate the statistical difference between responses of the 3D7 and 7G8 peptides. Proc Mixed of SAS 9.3 was used for this analysis.

## Results

### Predicted binding affinities of 3D7 and 7G8 AMA1 epitopes

The predicted binding affinities using the NetMHC algorithm ([12]) of the 3D7 and 7G8 AMA1 HLA-A*03 and B*58 epitopes were highly similar, suggesting that the single amino acid differences between the 3D7 and 7G8 A*03 and B*58 epitopes did not significantly affect predicted HLA-binding affinities. The predicted binding affinities of supertype 3D7 A*03 NSTC**R**FFVCK and 7G8 NSTC**N**FFVCK epitopes with A*11:01 were almost identical (119 nM and 111 nM), and in the typical range of MHC class I-restricted epitopes (< 500 nM). The predicted binding affinities of the supertype 3D7 B*58 KS**H**GKGYNW and 7G8 B*58 KS**R**GKGYNW epitopes were also similar with HLA-B*57:01 (11 nM, 15nM) and HLA-B*58:01 (9 nM, 28 nM).

### Ex vivo ELISpot and CD8+ T cell IFN-γ activities

#### HLA-A*03 subjects

**Fig 1:**
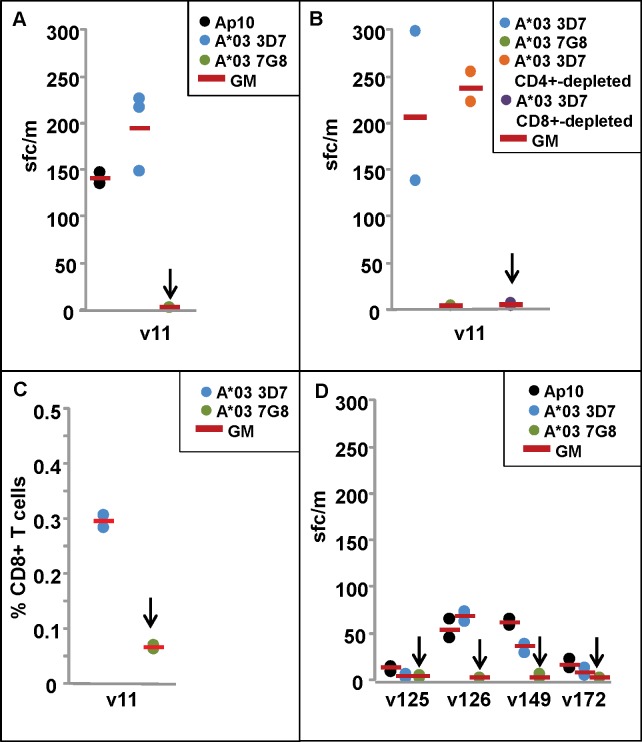
ELISpot and CD8+ T cell IFN-γ responses of DNA/HuAd5 and HuAd5 immunized subjects to *P*. *falciparum* strains 3D7 and 7G8 AMA1 A*03 protective epitopes. ELISpot and CD8+ T cell IFN-γ activities are shown in Panels A–D. **Panel A:** ELISpot IFN-γ response of the A*03 protected subject (v11) are positive with Ap8 and the 3D7 A*03 epitope but not the 7G8 epitope (arrow). **Panel B:** ELISpot activity of v11 is not affected by CD4+-depletion but is abolished after CD8+ depletion (arrow). **Panel C:** CD8+ T cell IFN-γ responses of v11 are much higher (p = 0.001) to the 3D7 epitope than to the 7G8 epitope (arrow). **Panel D:** ELISpot IFN-γ responses of two of four non-protected subjects from the HuAd5 trial were weakly positive with the 3D7 epitope but all four subjects were negative with the 7G8 epitope (arrows).

PBMCs of one protected HLA A*03 DNA/HuAd5-immunized subject (v11) were tested with Ap10 and the 3D7 and 7G8 HLA A*03 epitope peptides. All PBMCs were taken 12 weeks after CHMI. Ap10 recalled ELISpot IFN-γ responses from protected subject v11 that were similar to responses recalled by the 3D7 HLA A*03 epitope, whereas the 7G8 HLA A*03 epitope did not recall any responses (Table 2, Fig 1, Panel A).

**Table 2:** ELISpot IFN-γ activities of DNA/HuAd5-immunized and HuAd5-immunized subjects with AMA1 peptide pools and predicted 3D7 and 7G8 AMA1 epitopes.

					ELISpot IFN-γ (sfc/m)		
**Vaccine**	**Status**	**Subject**	**HLA**	Predicted 3D7 A*03	**Ap10**	3D7 A*03 epitope	7G8 A*03 epitope
				**affinity nM**			
DNA/HuAd5	P	v11	A*03	7	**139** (**134,138,146**)^1^	**193** (**148,216,226**)^1^	0 (0,0,0)^1^
HuAd5	Non-P	v125	A*03	7	12 (8,14,14)^2^	3 (2,2,6)^2^	3 (1,4,4)^2^
	Non-P	v126	A*01A03	36	**53 (44,64)**^2^	**67 (62,68,72)**^2^	2 (2,2,2)^2^
	Non-P	v149	A*03	7	**61 (58,60,64)**^2^	**34 (28,38,38)**^2^	2 (1,1,6)^2^
	Non-P	v172	A*03	227	15 (12,12,22)^2^	7 (4,6,12)^2^	0 (0,0,0)^2^
**Vaccine**	**Status**	**Subject**	**HLA**	Predicted 3D7 A*03	**Ap10**	3D7 A*03 epitope	7G8 A*03 epitope
				**affinity nM**			
DNA/HuAd5	P	v10	B*58	43	**88 (84,86,94)**^1^	**69 (56–86)**^1^	2 (1–4)^1^
	P	v18	B*58	21	**156 (136,162,174)**^1^	**132 (124,126,146)**^1^	2 (1,2,4)^1^
	Non-P	v15	B*58	43	26 (22,28,28)^1^	6 (2,6,14)^1^	0 (0,0,0)^1^
HuAd5	Non-P	v135	A*01	91	**65 (60,68,72)**^2^	**40 (34,42,46)**^2^	1 (0,0,2)^2^
	Non-P	v179	A*01	91	**65 (54,64,78)**^3^	**27 (20,28,36)**^3^	0 (0,0,0)^3^
	Non-P	v194^4^	B*58	21	**56 (54,56,58)**^3^	**27 (22,24,38)**^3^	0 (0,0,0)^3^

ELISpot IFN-γ activities are of protected (P) and non-protected subjects (Non-P) from the DNA/HuAd5 and HuAd5 trials with HLA-matched AMA1 peptide pools and predicted AMA1 epitopes. Activities are shown as the geometric mean and activities of individual replicates. Positive ELISpot activities are shown in bold. DNA/HuAd5 HLA A*03 protected subject (v11) had similar activities to the Ap10 peptide pool and the 3D7 A*03 epitope, but negative activity with the 7G8 A*03 epitope; HuAd5 trial HLA A*03 non-protected subjects had lower activities to Ap10 and the 3D7 epitope and were also negative with the 7G8 A*03 epitope; one of these (v125) was previously positive with Ap10 (Table 1) but was negative when re-tested, whereas a second subject (v172) was consistently negative with Ap10 (see Table 1). The DNA/HuAd5 HLA B*58 protected subjects (v10 and v18) also had similar activities to the Ap8 peptide pool and the 3D7 B*58 epitope, and were negative with the 7G8 epitope; One low responding DNA/HuAd5 subject (v15) and three HuAd5 HLA B*58 non-protected subjects (v136, v179 and v194^4^) had lower activities to Ap8 and the 3D7 B*58 epitope and were negative with the 7G8 epitope.

^1^DNA/HuAd5 12 weeks post-CHMI

^2^HuAd5 4 weeks post-CHMI

^3^HuAd5 12 weeks post-CHMI

^4^v194 was not sterilely protected but showed a significant delay to patency

In CD4+ and CD8+ ELISpot depletion studies with the 3D7 epitope, depletion of CD4+ T cells did not affect activities, whereas depletion of CD8+ T cells completely abolished recall activities (Table 3; Fig 1, Panel B).

**Table 3:** ELISpot IFN-γ depletion and CD8+ T cell IFN-γ activities of DNA/HuAd5-immunized and HuAd5-immunized subjects with AMA1 peptide pools and predicted 3D7 and 7G8 AMA1 epitopes.

				ELISpot IFN- γ				CD8+ T cell IFN-γ by ICS	
				3D7			7G8	3D7	7G8
Vaccine	HLA	Status	Subject	Non-depl.	CD4+ depl.	CD8 depl.	Non-depl.		
DNA/HuAd5	A*03	P	v11	**200**^1^	**231**^1^	2^1^	1^1^	**0.29**	**0.06**
				**(133,300)**	**(216, 247)**	(1,2)	(1,1)	**(0.28,0.30)**^2^	**(0.05,0.06)**^2^
	B*58	P	v10	**79**^1^	**30**^1^	4^1^	9^1^	**0.19**	0.028
				**(65,91,83)**	**(23,28,43)**	(1,7,7)	(7,7,13)	**(0.15,0.23)**^2^	(0.028,0.028)^2^
		P	v18	**105**^1^	**111**^1^	2^1^	2^1^	**0.43**	**0.08**
				**(98,105,112)**	**(98,108,130)**	(1,3,5)	(1,3,3)	**(0.39,0.46)**^2^	**(0.07,0.08)**^2^

Protected subjects from the DNA/HuAd5 trial were tested in ELISpot IFN-γ assays with the HLA-matched 3D7 and 7G8 epitopes after CD4+ and CD8+ T cell depletions (see Methods), and in ICS assays to confirm CD8+ T cell IFN-γ activities. Positive activities are shown in bold. The HLA A*03 protected subject (v11) retained full activity after CD4+ T cell depletion, but CD8+ T cell depletion completely removed activity; v11 had no activity with the 7G8 epitope that was unaffected by CD4+ and CD8+ depletions; CD8+ T cell IFN-γ activity assessed by ICS was approximately 5-times higher to the 3D7 epitope than the 7G8 epitope. Activity of the HLA B*58 protected subject (v10) was reduced approximately 3-fold after CD4+ T cell depletion, but CD8+ T cell depletion completely abolished activity; v10 had no activity with the 7G8 epitope that was unaffected by CD4+ and CD8+ depletions; CD8+ T cell IFN-γ activity was directed to the 3D7 and not the 7G8 epitope. Activity of the second HLA B*58 protected subject (v18) was unaffected by CD4+ T cell depletion and was completely abolished by CD8+ T cell depletion; v18 had no activity with the 7G8 epitope that was unaffected by CD4+ and CD8+ depletions; CD8+ T cell IFN-γ was predominantly directed to the 3D7 epitope and was significantly (p = 0.001) higher than the 7G8 epitope.

^1^DNA/HuAd5 12 weeks post-CHMI

^2^DNA/HuAd5 pre-CHMI

The 3D7 epitope-specificity of CD8+ T cells was confirmed in ICS assays, using pre-CHMI PBMCs; CD8+ T cell IFN-γ responses of v11 to the 3D7 HLA A*03 epitope were significantly higher (p = 0.001) than to the 7G8 HLA A*03 epitope (Table 2, Fig 1, Panel C). CD4+ T cell IFN-γ responses were low and did not reach positive values.

This 3D7 vaccine strain-specificity was also shown using PBMCs from HuAd5-immunized non-protected subjects that expressed different HLA alleles that were also predicted to bind to the HLA A*03 epitope: two of these four subjects were positive:v126, expressing HLA A:30:01 that is A*01A03 supertype; and v149, expressing HLA A*11:01 that is A*03 supertype; each had weakly positive ELISpot IFN-γ responses to Ap10 and the 3D7 A*03 epitope that were lower than v11, whereas the 7G8 A*03 epitope did not recall ELISpot IFN-γ responses from any of these subjects (Table 2; Fig 1, panel D); two other HuAd5-immunized subjects expressing A*01A*03 or A*03 supertypes that were predicted to bind to the 3D7 HLA A*03 epitope were negative with both 3D7 and 7G8 HLA A*03 epitopes (Table 2; Fig 1, panel D),.

#### HLA-B*58 subjects

**Fig 2:**
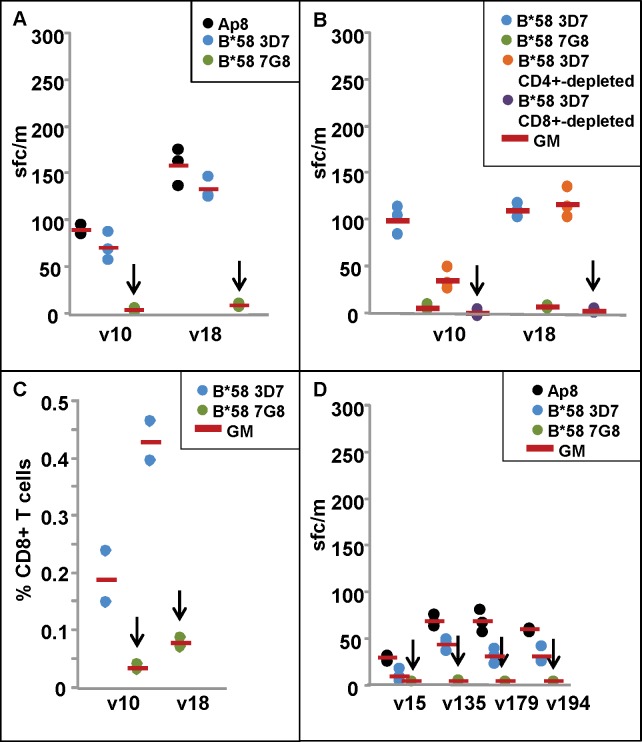
ELISpot and CD8+ T cell IFN-γ responses of DNA/HuAd5 and HuAd5 immunized subjects to *P*. *falciparum* strains 3D7 and 7G8 AMA1 B*58 protective epitopes. ELISpot and CD8+ T cell IFN-γ activities are shown in Panels A–D. **Panel A:** ELISpot IFN-γ responses of the B*58 protected subjects (v10, v18) are positive with Ap10 and the 3D7 B*58 epitope but not 7G8 epitopes (arrows). **Panel B:** ELISpot activity of v10 is reduced but still remains positive after CD4+-depletion, but is abolished after CD8+-depletion (arrow); activity of v18 is unaffected by CD4+-depletion but is abolished after CD8+-depletion (arrow). **Panel C:** CD8+ T cell responses of v10 and v18 are much higher (p = 0.001) against the 3D7 B*58 epitope rather than the 7G8 B*58 epitope (arrows). **Panel D:** ELISpot IFN-γ response of DNA/HuAd5 non-protected B*58 subject (v15) was negative and non-protected v194 was weakly positive with the 3D7 B*58 epitope; two non-protected subjects from the HuAd5 trial that express A*01 (v135, v179) were weakly positive with the 3D7 B*58 epitope; all these subjects were negative with the 7G8 B*58 epitope (arrows).

PBMCs from two protected HLA B*58 subjects (v10, v18), and one non-protected HLA B*58 (v15) who had lower activities pre-CHMI (Table 1) from DNA/HuAd5-immunized subjects were tested with Ap8 and 3D7 and 7G8 HLA B*58 epitope peptides. All PBMCs were taken 12 weeks after CHMI. Ap8 recalled ELISpot IFN-γ responses from protected v10 and v18 that were similar to responses recalled by the 3D7 HLA B*58 epitope, whereas the 7G8 HLA B*58 epitope did not recall any responses (Table 2; Fig 2, Panel A). The non-protected low responding DNA/HuAd5 subject v15 was negative in all assays.

In CD4+ and CD8+ T cell ELISpot depletion studies in DNA/HuAd5-immunized subjects with the 3D7 B*58 epitope, depletion of CD4+ T cells reduced activity of v10 approximately three-fold although activity remained positive, but did not affect activity of v18; however, depletion of CD8+ T cells completely abolished activities of v10 and v18 (Table 3, Fig 2, Panel B).

The 3D7 B*58 epitope-specificity of CD8+ T cells was confirmed in ICS assays, using pre-CHMI PBMCs; CD8+ T cell IFN-γ responses of v10 and v18 to the 3D7 HLA B*58 epitope were five-six-fold higher (p = 0.001) than to the 7G8 B*58 epitope (Table 3, Fig 2, Panel C).

This 3D7 vaccine strain-specificity was also shown using PBMCs from HuAd5-immunized subjects; one HLA B*58 subject (v194), who was not sterilely protected but showed a significant delay to parasitemia [2], was also positive with the HLA B*58 epitope but not the 7G8 HLA B*58 epitope; and two non-protected subjects (v135 and v179, each expressing HLA A*32:01 that is A*01 supertype) who were weakly positive with the 3D7 B*58 (recognized through a different HLA, A*01), whereas the 7G8 B*58 epitope did not recall ELISpot IFN-γ responses from either of these subjects (Table 2; Fig 2, Panel D).

## Discussion

The major conclusion from these studies is that immunization with the heterologous DNA prime/HuAd5 vaccine, and immunization with HuAd5 alone expressing 3D7 AMA1 induced vaccine strain-specific CD8+ T cell responses to the dimorphic A*03 and B*58 epitopes found in strain 3D7 that were not recalled by the 7G8 A*03 and B*58 variant epitopes. This result was unexpected since the NetMHC algorithm [4] predicted that the 7G8 epitopes had similar predicted binding affinities as the 3D7 epitopes and we therefore expected that the 7G8 variant epitopes would also recall activities from the 3D7-immunized subjects. We previously used the same predictive algorithm [4] to predict 3D7 HLA A*03 and B*58 epitopes that recalled CD8+ T cells IFN-γ responses from the DNA/HuAd5 and HuAd5 3D7 AMA1-immunized subjects [3]. Since the similar predicted binding affinities of the 3D7 and 7G8 epitopes were similar, these outcomes emphasize the need for functional assays of epitope activities in design of malaria vaccines.

The similar predicted binding affinities of the 3D7 and 7G8 epitopes also suggested that both allelic sequences were able to bind to A*03 and B*58 MHC molecules. Interestingly, the 3D7 HLA B*58 epitope KS**H**GKGYNW was predicted to have similar binding affinities to the 7G8 HLA B*58 supertype, and yet the 7G8 HLA B*58 epitope failed to also recall activities from two subjects in the HuAd5 trial expressing HLA B*58.

HLA supertype peptide B pocket (aa in position 2) and F pocket (aa in position 9) specificities for A*03 epitope STC**R**FFVCK are small and aliphatic: Threonine (T) and basic: Lysine (K), and for B*58 epitope KS**H**GKGYNW are small: Serine (S) and large and hydrophobic/aromatic: Tryptophan (W) [13]. These aa are conserved in both 3D7 and 7G8 strains [13] probably accounting for the similar predicted binding affinities to HLA A*03 and B*58 [13]. Since we have not directly measured binding affinities of the 3D7 and 7G8 epitopes to HLA A*03 and B*58 we can only hypothesize that the 3D7 and 7G8 epitopes bind to each HLA supertype, based on this predictive algorithm [9]. Based on this assumption, we suggest there may be two other likely interpretations of these results.

Firstly, it is possible that the lack of recall activities of the 7G8 epitopes is due to impaired T cell receptor (TCR) recognition of the MHC-peptide complex. TCR α- and β- chains bind to the MHC-peptide complex to form a tri-molecular MHC-peptide-TCR complex that activates the immune system [14–16]. Structural analyses of TCR:peptide:MHC complexes [17] have revealed that amino acids between the two anchor residues are most frequently in direct contact with the TCR. Single residue mutations in the epitope contact residues of a TCR have been shown to be sufficient to abrogate recognition [18–20]. Since the 3D7 and 7G8 dimorphic variations occur between these anchor residues on position 4 (A*03) and position 3 (B*58), it is possible that these variations in 7G8 abrogate recognition by TCRs induced against the 3D7 epitope, supporting this first hypothesis. Moreover, studies of escape mutations in influenza virus have shown that some mutations eliminate binding to MHC, while other mutations retain the ability to bind to MHC but are no longer recognized by the TCR recognizing the wild type peptide [21, 22]. In chronic HIV infections, escape mutations that abrogate MHC binding completely are found more frequently, as such escape mutations provide the pathogen with permanent protection from T cell recognition, while pathogens with TCR escape mutations are still vulnerable to new T cell populations arising that recognize the mutation [23]. Any mutation that abrogates recognition of a pathogen by vaccine induced immune cells without interfering with overall pathogen fitness has an evolutionary advantage and can lead to reduced vaccine efficacy.

A second interpretation is that the 7G8 AMA1 variant epitopes may act as altered peptide ligands (APLs) that contain single or multiple aa changes that affect immunogenicity or recognition by pre-existing (such as vaccine-induced) immune responses [24–26]. *P*. *falciparum* CSP T cell regions contain highly polymorphic CD4+ and CD8+ T cell epitopes, and only two variants bind HLA-B35; naturally induced CD8+ T cells to these two variants are not cross-reactive, suggesting the influence of local HLA on malaria antigenic variation [27]. Such variant amino acids have a different orientation when binding to the HLA peptide binding groove, and cause the TCR-binding amino acids to remain buried and unavailable for binding to the TCR as earlier suggested for myelin binding to HLA-DR2b [28] or peptide antagonism of NK cell activation [29].

Antigenic diversity of parasite proteins leads to immune evasion due to a loss of recognition by antibodies and immune T cells [30] including AMA1 [31]. Immunization with one AMA1 allele may not induce antibodies that protect against different alleles [32], and multi-allele AMA vaccines may be required [31] particularly to the pentavalent epitope [33] and a multi-allele AMA1 vaccine approach would induce broad coverage against diverse alleles [7]. Recently, the ChAd63/MVA vaccine containing 3D7 and FVO AMA1 proteins from two strains (3D7 and FVO) elicited recall activation of T cell responses to both variants but with a bias to the 3D7/MAD20 allele [34] suggesting that APLs within AMA1 may have affected antigenic priming and recall activation T cells, or may have resulted from a lack of class I epitopes in the FVO strain. However, APLs within malaria antigen surface antigen-1 (MSP-1) inhibited IFN-γ responses of naturally-infected Gambian subjects and impaired priming of T cells from malaria naïve subjects [35], and by presentation of APLs on the same antigen-presenting cell [36]. Further experiments are required to establish whether APLs are responsible for the lack of recall activities by the 7G8 AMA1 peptides in this study.

Another heterologous prime-boost strategy using chimpanzee adenovirus ChAd63-prime/ modified vaccine virus Ankara (MVA) boost and *P*. *falciparum* TRAP antigen induced similar levels of sterile protection also associated with CD8+ T cell IFN-γ responses to TRAP [37]. HLA A*03 subjects recognized discrete areas of T9/96 TRAP suggesting that this regimen may also have induced CD8+ T cells recognizing TRAP class I epitopes [38].

We have previously demonstrated that other 3D7 A*01, A*02, B*08 and B*44 predicted class 1-restricted epitopes in AMA1 are polymorphic and recalled ELISpot IFN-γ activities (predominantly CD8+ T cells) from HLA-matched subjects immunized with the HuAd5 vaccine [39], but at that time, A*03 or B*58 epitopes were not tested, nor did we evaluate whether variant epitopes also recalled responses from these subjects [39]. However, we did later evaluate nine class I-restricted 3D7 CSP epitopes predicted by NetMHC for recall responses from the HuAd5-immunized subjects [40]. One of these CSP epitopes recalled activities from HLA A*23:01 (HLA A*24 supertype) and HLA A*29:02 (HLA A*01A24 supertype) subjects, and one CSP epitope recalled responses from A*23:01 (HLA A24 supertype), A*29:02 (HLA A01A24 supertype) and B*15:03 (HLA B27 supertype) [40]. Earlier analyses have suggested that malaria antigens are highly promiscuous and bind to more than one HLA allele [41] which is consistent with other work that different alleles overlap in their peptide-binding properties [13, 42]. Since both CSP and AMA1 class I-restricted epitopes, either predicted or evaluated in functional assays, are polymorphic, it is unclear whether AMA1 T cell-based vaccines will elicit responses to multiple HLA alleles, or whether a multi-allele AMA1 vaccine would be required. A potential advantage of combining variants is that this may hinder development of escape mutants in natural infection containing further polymorphism of epitopes not included in the vaccine.

The most effective malaria vaccines are whole sporozoites that are thought to target multiple antigens [43]. The more antigens and epitopes that are targeted by vaccine induced immunity, the more difficult it becomes for a pathogen to escape. Thus in the development of subunit malaria vaccines, it is desirable to include either multiple antigens or epitopes that cover the genetic diversity of endemic strains. Additionally, subunit malaria vaccines allow focusing the vaccine induced immune response on antigens and epitopes that have a higher fitness cost for the pathogen to mutate.

### Conclusions

This study highlights the need to assess the ability of vaccines to induce immune responses and protection across different strains, and that functional assays are critical for their development. The fact that both epitopes in AMA1 that we had identified to be targets of protective immunity were not cross-reactive also raises the question of whether alternative antigens that show less strain-to-strain variability can be identified.

## References

[1] Chuang I, Sedegah M, Cicatelli S, Spring M, Polhemus M, Tamminga C, et al DNA prime/Adenovirus boost malaria vaccine encoding P. falciparum CSP and AMA1 induces sterile protection associated with cell-mediated immunity. PLoS One. 2013;8:e55571 10.1371/journal.pone.0055571 23457473 PMC3573028

[2] Tamminga C, Sedegah M, Maiolatesi S, Fedders C, Reyes S, Reyes A, et al Human adenovirus 5-vectored Plasmodium falciparum NMRC-M3V-Ad-PfCA vaccine encoding CSP and AMA1 is safe, well-tolerated and immunogenic but does not protect against controlled human malaria infection. Hum Vaccin Immunother. 2013;9:2165–77. 10.4161/hv.24941 23899517 PMC3906401

[3] Sedegah M, Hollingdale MR, Farooq F, Ganeshan H, Belmonte M, Kim Y, et al Sterile Immunity to Malaria after DNA Prime/Adenovirus Boost Immunization Is Associated with Effector Memory CD8+T Cells Targeting AMA1 Class I Epitopes. PLoS One. 2014;9:e106241 10.1371/journal.pone.0106241 25211344 PMC4161338

[4] Lundegaard C, Lamberth K, Harndahl M, Buus S, Lund O, Nielsen M. NetMHC-3.0: accurate web accessible predictions of human, mouse and monkey MHC class I affinities for peptides of length 8–11. Nucleic Acids Res. 2008;36:W509–12. 10.1093/nar/gkn202 18463140 PMC2447772

[5] Barry AE, Arnott A. Strategies for designing and monitoring malaria vaccines targeting diverse antigens. Front Immunol. 2014;5:359 10.3389/fimmu.2014.00359 25120545 PMC4112938

[6] Remarque EJ, Faber BW, Kocken CH, Thomas AW. Apical membrane antigen 1: a malaria vaccine candidate in review. Trends Parasitol. 2008;24:74–84. 10.1016/j.pt.2007.12.002 18226584

[7] Drew DR, Hodder AN, Wilson DW, Foley M, Mueller I, Siba PM, et al Defining the antigenic diversity of Plasmodium falciparum apical membrane antigen 1 and the requirements for a multi-allele vaccine against malaria. PLoS One. 2012;7:e51023 10.1371/journal.pone.0051023 23227229 PMC3515520

[8] Sedegah M, Tamminga C, McGrath S, House B, Ganeshan H, Lejano J, et al Adenovirus 5-vectored P. falciparum Vaccine Expressing CSP and AMA1. Part A: Safety and Immunogenicity in Seronegative Adults. PLoS One. 2011;6:e24586 10.1371/journal.pone.0024586 22003383 PMC3189181

[9] Nielsen M, Lundegaard C, Blicher T, Lamberth K, Harndahl M, Justesen S, et al NetMHCpan, a method for quantitative predictions of peptide binding to any HLA-A and -B locus protein of known sequence. PLoS One. 2007;2:e796 10.1371/journal.pone.0000796 17726526 PMC1949492

[10] Wang R, Epstein J, Baraceros FM, Gorak EJ, Charoenvit Y, Carucci DJ, et al Induction of CD4(+) T cell-dependent CD8(+) type 1 responses in humans by a malaria DNA vaccine. Proc Natl Acad Sci U S A. 2001;98:10817–22. 10.1073/pnas.181123498 11526203 PMC58557

[11] Stewart VA, McGrath SM, Dubois PM, Pau MG, Mettens P, Shott J, et al Priming with an adenovirus 35-circumsporozoite protein (CS) vaccine followed by RTS,S/AS01B boosting significantly improves immunogenicity to Plasmodium falciparum CS compared to that with either malaria vaccine alone. Infect Immun. 2007;75:2283–90. 10.1128/IAI.01879-06 17307942 PMC1865796

[12] Lundegaard C, Lund O, Nielsen M. Accurate approximation method for prediction of class I MHC affinities for peptides of length 8, 10 and 11 using prediction tools trained on 9mers. Bioinformatics. 2008;24:1397–8. 10.1093/bioinformatics/btn128 18413329

[13] Sidney J, Peters B, Frahm N, Brander C, Sette A. HLA class I supertypes: a revised and updated classification. BMC Immunol. 2008;9:1 10.1186/1471-2172-9-1 18211710 PMC2245908

[14] Garcia KC, Degano M, Stanfield RL, Brunmark A, Jackson MR, Peterson PA, et al An alphabeta T cell receptor structure at 2.5 A and its orientation in the TCR-MHC complex. Science. 1996;274:209–19. 10.1126/science.274.5285.209 8824178

[15] Hennecke J, Carfi A, Wiley DC. Structure of a covalently stabilized complex of a human alphabeta T-cell receptor, influenza HA peptide and MHC class II molecule, HLA-DR1. EMBO J. 2000;19:5611–24. 10.1093/emboj/19.21.5611 11060013 PMC305780

[16] Marrack P, Scott-Browne JP, Dai S, Gapin L, Kappler JW. Evolutionarily conserved amino acids that control TCR-MHC interaction. Annu Rev Immunol. 2008;26:171–203. 10.1146/annurev.immunol.26.021607.090421 18304006 PMC3164820

[17] Hawse WF, De S, Greenwood AI, Nicholson LK, Zajicek J, Kovrigin EL, et al TCR scanning of peptide/MHC through complementary matching of receptor and ligand molecular flexibility. J Immunol. 2014;192:2885–91. 10.4049/jimmunol.1302953 24523505 PMC3992338

[18] Pircher H, Moskophidis D, Rohrer U, Burki K, Hengartner H, Zinkernagel RM. Viral escape by selection of cytotoxic T cell-resistant virus variants in vivo. Nature. 1990;346:629–33. 10.1038/346629a0 1696684

[19] Bermudez A, Calderon D, Moreno-Vranich A, Almonacid H, Patarroyo MA, Poloche A, et al Gauche(+) side-chain orientation as a key factor in the search for an immunogenic peptide mixture leading to a complete fully protective vaccine. Vaccine. 2014;32:2117–26. 10.1016/j.vaccine.2014.02.003 24582630

[20] Patarroyo ME, Bermudez A, Alba MP, Vanegas M, Moreno-Vranich A, Poloche LA, et al IMPIPS: the immune protection-inducing protein structure concept in the search for steric-electron and topochemical principles for complete fully-protective chemically synthesised vaccine development. PLoS One. 2015;10:e0123249 10.1371/journal.pone.0123249 25879751 PMC4400017

[21] Valkenburg SA, Gras S, Guillonneau C, Hatton LA, Bird NA, Twist KA, et al Preemptive priming readily overcomes structure-based mechanisms of virus escape. Proc Natl Acad Sci U S A. 2013;110:5570–5. 10.1073/pnas.1302935110 23493558 PMC3619348

[22] Valkenburg SA, Quinones-Parra S, Gras S, Komadina N, McVernon J, Wang Z, et al Acute emergence and reversion of influenza A virus quasispecies within CD8+ T cell antigenic peptides. Nat Commun. 2013;4:2663 10.1038/ncomms3663 24173108

[23] Bronke C, Almeida CA, McKinnon E, Roberts SG, Keane NM, Chopra A, et al HIV escape mutations occur preferentially at HLA-binding sites of CD8 T-cell epitopes. AIDS. 2013;27:899–905. 10.1097/QAD.0b013e32835e1616 23276808 PMC3818524

[24] Plebanski M, Lee EA, Hill AV. Immune evasion in malaria: altered peptide ligands of the circumsporozoite protein. Parasitology. 1997;115 Suppl:S55–66. 10.1017/s0031182097002035 9571691

[25] Flanagan KL, Wilson KL, Plebanski M. Polymorphism in liver-stage malaria vaccine candidate proteins: immune evasion and implications for vaccine design. Expert Rev Vaccines. 2016;15:389–99. 10.1586/14760584.2016.1125785 26610026

[26] Wilson KL, Xiang SD, Plebanski M. A Model to Study the Impact of Polymorphism Driven Liver-Stage Immune Evasion by Malaria Parasites, to Help Design Effective Cross-Reactive Vaccines. Front Microbiol. 2016;7:303 10.3389/fmicb.2016.00303 27014226 PMC4786561

[27] Gilbert SC, Plebanski M, Gupta S, Morris J, Cox M, Aidoo M, et al Association of malaria parasite population structure, HLA, and immunological antagonism. Science. 1998;279:1173–7. 10.1126/science.279.5354.1173 9469800

[28] Mantzourani ED, Platts JA, Brancale A, Mavromoustakos TM, Tselios TV. Molecular dynamics at the receptor level of immunodominant myelin basic protein epitope 87–99 implicated in multiple sclerosis and its antagonists altered peptide ligands: triggering of immune response. J Mol Graph Model. 2007;26:471–81. 10.1016/j.jmgm.2007.02.004 17392002

[29] Fadda L, Borhis G, Ahmed P, Cheent K, Pageon SV, Cazaly A, et al Peptide antagonism as a mechanism for NK cell activation. Proc Natl Acad Sci U S A. 2010;107:10160–5. 10.1073/pnas.0913745107 20439706 PMC2890497

[30] Kirkman LA, Deitsch KW. Recombination and Diversification of the Variant Antigen Encoding Genes in the Malaria Parasite Plasmodium falciparum. Microbiol Spectr. 2014;2 10.1128/microbiolspec.MDNA3-0022-2014 26104446

[31] Terheggen U, Drew DR, Hodder AN, Cross NJ, Mugyenyi CK, Barry AE, et al Limited antigenic diversity of Plasmodium falciparum apical membrane antigen 1 supports the development of effective multi-allele vaccines. BMC Med. 2014;12:183 10.1186/s12916-014-0183-5 25319190 PMC4212128

[32] Thera MA, Doumbo OK, Coulibaly D, Laurens MB, Ouattara A, Kone AK, et al A field trial to assess a blood-stage malaria vaccine. N Engl J Med. 2011;365:1004–13. 10.1056/NEJMoa1008115 21916638 PMC3242358

[33] Coley AM, Parisi K, Masciantonio R, Hoeck J, Casey JL, Murphy VJ, et al The most polymorphic residue on Plasmodium falciparum apical membrane antigen 1 determines binding of an invasion-inhibitory antibody. Infect Immun. 2006;74:2628–36. 10.1128/IAI.74.5.2628-2636.2006 16622199 PMC1459722

[34] Elias SC, Collins KA, Halstead FD, Choudhary P, Bliss CM, Ewer KJ, et al Assessment of immune interference, antagonism, and diversion following human immunization with biallelic blood-stage malaria viral-vectored vaccines and controlled malaria infection. J Immunol. 2013;190:1135–47. 10.4049/jimmunol.1201455 23293353 PMC3672846

[35] Lee EA, Flanagan KL, Minigo G, Reece WH, Bailey R, Pinder M, et al Dimorphic Plasmodium falciparum merozoite surface protein-1 epitopes turn off memory T cells and interfere with T cell priming. Eur J Immunol. 2006;36:1168–78. 10.1002/eji.200526010 16619284

[36] Plebanski M, Lee EA, Hannan CM, Flanagan KL, Gilbert SC, Gravenor MB, et al Altered peptide ligands narrow the repertoire of cellular immune responses by interfering with T-cell priming. Nat Med. 1999;5:565–71. 10.1038/8444 10229235

[37] Ewer KJ, O'Hara GA, Duncan CJ, Collins KA, Sheehy SH, Reyes-Sandoval A, et al Protective CD8+ T-cell immunity to human malaria induced by chimpanzee adenovirus-MVA immunisation. Nat Commun. 2013;4:2836 10.1038/ncomms3836 24284865 PMC3868203

[38] Hodgson SH, Juma E, Salim A, Magiri C, Kimani D, Njenga D, et al Evaluating controlled human malaria infection in Kenyan adults with varying degrees of prior exposure to Plasmodium falciparum using sporozoites administered by intramuscular injection. Front Microbiol. 2014;5:686 10.3389/fmicb.2014.00686 25566206 PMC4264479

[39] Sedegah M, Kim Y, Peters B, McGrath S, Ganeshan H, Lejano J, et al Identification and localization of minimal MHC-restricted CD8+ T cell epitopes within the Plasmodium falciparum AMA1 protein. Malar J. 2011;9:241 10.1186/1475-2875-9-241 20735847 PMC2939619

[40] Sedegah M, Kim D, Ganeshan H, Huang J, Belmonte M, Abot E, et al Identification of minimal human MHC-restricted CD8+ T-cell epitopes within the Plasmodium falciparum circumsporozoite protein (CSP). Malar J. 2013 10.1186/1475-2875-12-185 23738590 PMC3683343

[41] Doolan DL, Southwood S, Chesnut R, Appella E, Gomez E, Richards A, et al HLA-DR-promiscuous T cell epitopes from Plasmodium falciparum pre-erythrocytic-stage antigens restricted by multiple HLA class II alleles. J Immunol. 2000;165:1123–37. 10.4049/jimmunol.165.2.1123 10878392

[42] Sette A, Sidney J. Nine major HLA class I supertypes account for the vast preponderance of HLA-A and -B polymorphism. Immunogenetics. 1999;50:201–12. 10.1007/s002510050594 10602880

[43] Seder RA, Chang LJ, Enama ME, Zephir KL, Sarwar UN, Gordon IJ, et al Protection against malaria by intravenous immunization with a nonreplicating sporozoite vaccine. Science. 2013;341:1359–65. 10.1126/science.1241800 23929949

