# Correction: Vaccine Strain-Specificity of Protective HLA-Restricted Class 1 *P*. *falciparum* Epitopes

**DOI:** 10.1371/journal.pone.0168952

**Published:** 2016-12-20

**Authors:** Martha Sedegah, Bjoern Peters, Michael R. Hollingdale, Harini D. Ganeshan, Jun Huang, Fouzia Farooq, Maria N. Belmonte, Arnel D. Belmonte, Keith J. Limbach, Carter Diggs, Lorraine Soisson, Ilin Chuang, Eileen D. Villasante

The legends to Figs 1 and 2 have errors in the Panel A section. The authors have provided corrected versions here.

**Fig 1:**
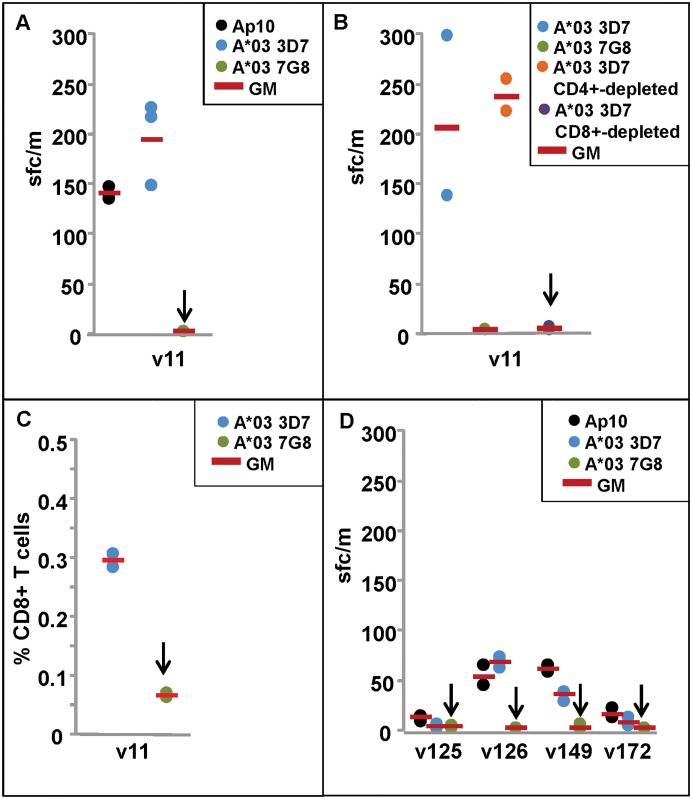
ELISpot and CD8+ T cell IFN-γ responses of DNA/HuAd5 and HuAd5 immunized subjects to *P*. *falciparum* strains 3D7 and 7G8 AMA1 A*03 protective epitopes. ELISpot and CD8+ T cell IFN-γ activities are shown in Panels A–D. **Panel A**: ELISpot IFN-γ response of the A*03 protected subject (v11) are positive with Ap10 and the 3D7 A*03 epitope but not the 7G8 epitope (arrow). **Panel B**: ELISpot activity of v11 is not affected by CD4+-depletion but is abolished after CD8+ depletion (arrow). **Panel C**: CD8+ T cell IFN-γ responses of v11 are much higher (p = 0.001) to the 3D7 epitope than to the 7G8 epitope (arrow). **Panel D**: ELISpot IFN-γ responses of two of four non-protected subjects from the HuAd5 trial were weakly positive with the 3D7 epitope but all four subjects were negative with the 7G8 epitope (arrows).

**Fig 2:**
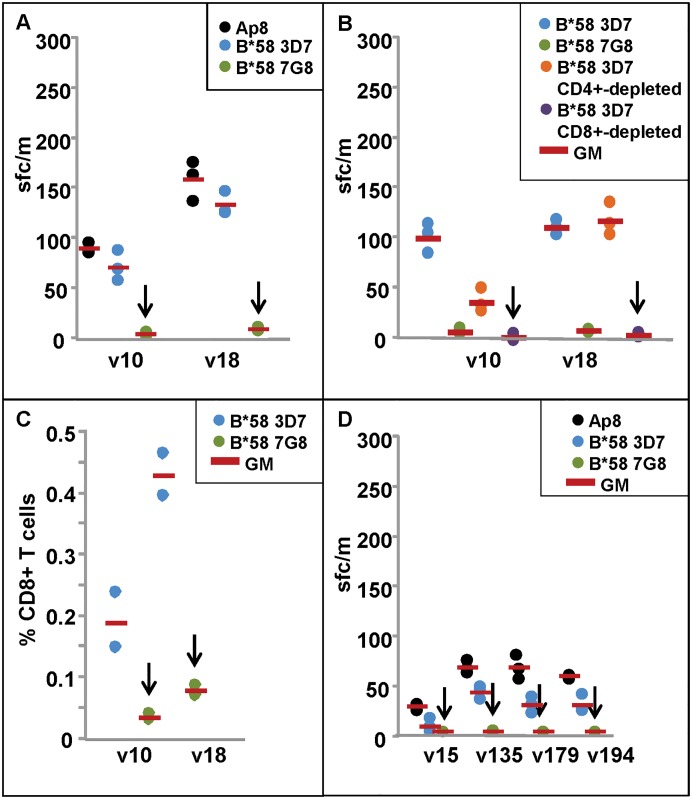
ELISpot and CD8+ T cell IFN-γ responses of DNA/HuAd5 and HuAd5 immunized subjects to *P*. *falciparum* strains 3D7 and 7G8 AMA1 B*58 protective epitopes. ELISpot and CD8+ T cell IFN-γ activities are shown in Panels A–D. **Panel A**: ELISpot IFN-γ responses of the B*58 protected subjects (v10, v18) are positive with Ap8 and the 3D7 B*58 epitope but not 7G8epitopes (arrows). **Panel B**: ELISpot activity of v10 is reduced but still remains positive after CD4+-depletion, but is abolished after CD8+-depletion (arrow); activity of v18 is unaffected by CD4+-depletion but is abolished after CD8+-depletion (arrow). **Panel C**: CD8+ T cell responses of v10 and v18 are much higher (p = 0.001) against the 3D7 B*58 epitope rather than the 7G8 B*58 epitope (arrows). **Panel D**: ELISpot IFN-γ response of DNA/HuAd5 non-protected B*58 subject (v15) was negative and non-protected v194 was weakly positive with the 3D7 B*58 epitope; two non-protected subjects from the HuAd5 trial that express A*01 (v135, v179) were weakly positive with the 3D7 B*58 epitope; all these subjects were negative with the 7G8 B*58 epitope (arrows).
